# Recent Progresses and Development of Advanced Atomic Layer Deposition towards High-Performance Li-Ion Batteries

**DOI:** 10.3390/nano7100325

**Published:** 2017-10-14

**Authors:** Wei Lu, Longwei Liang, Xuan Sun, Xiaofei Sun, Chen Wu, Linrui Hou, Jinfeng Sun, Changzhou Yuan

**Affiliations:** School of Material Science and Engineering, University of Jinan, Jinan 250022, China; ym0808@sohu.com (W.L.); llw198611@163.com (L.L.); 13583131953@163.com (X.S.); Asunxf_ME@163.com (X.S.); zzwuchen@126.com (C.W.); houlr629@163.com (L.H.); sunjinfeng2010@163.com (J.S.)

**Keywords:** atomic layer deposition, electrode materials, solid-state electrolytes, electrode-electrolyte sur-/interfaces, Li-ion batteries

## Abstract

Electrode materials and electrolytes play a vital role in device-level performance of rechargeable Li-ion batteries (LIBs). However, electrode structure/component degeneration and electrode-electrolyte sur-/interface evolution are identified as the most crucial obstacles in practical applications. Thanks to its congenital advantages, atomic layer deposition (ALD) methodology has attracted enormous attention in advanced LIBs. This review mainly focuses upon the up-to-date progress and development of the ALD in high-performance LIBs. The significant roles of the ALD in rational design and fabrication of multi-dimensional nanostructured electrode materials, and finely tailoring electrode-electrolyte sur-/interfaces are comprehensively highlighted. Furthermore, we clearly envision that this contribution will motivate more extensive and insightful studies in the ALD to considerably improve Li-storage behaviors. Future trends and prospects to further develop advanced ALD nanotechnology in next-generation LIBs were also presented.

## 1. Introduction

**Figure 1:**
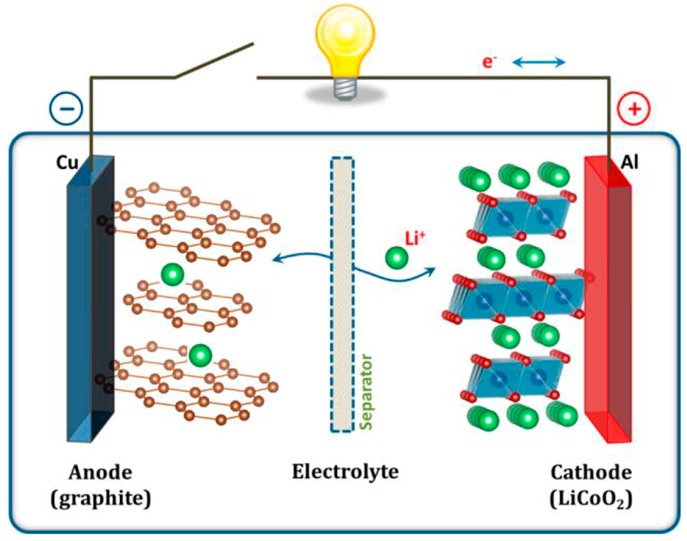
Schematic illustration for working mechanism of rechargeable lithium-ion batteries (LIBs). Reprinted with permission from Ref. [1] @copyright 2013 American Chemical Society.

Nowadays, the wide application of electric vehicles (EV) and hybrid EV may be one of the best approaches to reduce the continuous consumption of traditional fossil fuels, which can alleviate the deepening crises about climate change and air pollution. The critical challenge for high-powered electric transportation is the exploitation of high-performance energy storage devices with high power and energy densities, and high reliability [1,2,3]. Lithium-ion batteries (LIBs) thus have been regarded as one of ideal power supplies, thanks to their overwhelmingly high volumetric/gravimetric energy densities along with other superiorities including affordable price, long cycle life, and environmental friendliness [4,5,6]. Typically, as shown in Figure 1, a LIB cell consists of a lithium metal oxide cathode (positive electrode, e.g., LiCoO_2_), a carbon anode (negative electrode, e.g., graphite), an electronically insulating separator, and an ionically conductive Li^+^-containing electrolyte [1]. During the charging process, Li^+^ ions commonly migrate from the cathode structure, transport through the electrolyte, and finally intercalate into the anode. Concurrently, the electrons flow from the cathode to anode through an outer circuit. Upon a reversible discharging process occurring, so-called the rocking chair batteries, LIBs rely on the shuttling of lithium-ions back and forth between the two electrodes during charge-discharge cycles. Consequently, the employed electrode materials intensely govern both cell voltage and specific capacity of LIBs. 

To this end, electrode materials to fulfill high safety, large capacity, and long cycle lifetime are being developed by collaborative efforts in universities, national labs and industries around the world [7,8]. As widely demonstrated, conventional layered Li_1−*x*_CoO_2_ (LCO) is distressfully trapped in the phase distortion from monoclinic to hexagonal phase during the repeated charge-discharge process particularly when *x* > 0.5, which greatly limits the practical capacities of the LCO cathodes with merely half of its theoretical capacity [9,10]. Although Ni-based cathodes, such as LiNi_1/3_Mn_1/3_Co_1/3_O_2_ (NMC333), LiNi_0.8_Mn_0.1_Co_0.1_O_2_ (NMC811), LiNi_0.6_Mn_0.2_Co_0.2_O_2_ (NMC662), LiNi_0.5_Mn_0.3_Co_0.2_O_2_ (NMC532), and so on, offer an integration of high reversible capacity, high operating voltage and acceptable chemical stability. However, they still seriously suffer from the interfacial structural degeneration (layered-spinel-(rock-salt)) and are terribly sensitive to the humidity (Li-containing impurities deposited) [11,12,13]. Within the group of layered cathodes, Li-rich layered oxides (LLOs) have attracted plenty of attention owing to their large capacities (~250–300 mAh·g^−1^). Unfortunately, the oxygen loss from the lattice during the initial charge and the incident migration of the manganese occurs from the transition-metal plane to the lithium plane. It leads to high irreversible capacity loss in the first cycle, substantial voltage decay upon cycling and inferior rate capability, which enormously hinders the commercial application of the LLOs in LIBs [14,15]. As for spinel cathodes, such as LiMn_2_O_4_, it is also tremendously restricted in consequence by the limited discharge capacities (~120 mAh·g^−1^) and unsatisfactory high-temperature electrochemical properties caused by the critical issues stemming from the Jahn-Teller distortion, manganese dissolution and oxygen deficiency [16,17]. Equally, the anode materials employed in LIBs also play a significant role, while it is still confronted with various troubles. As is well known, Li metal was once regarded as one of the most promising anode materials for beyond Li-ion batteries owing to its low electrochemical potential and high theoretical specific capacity (~3861 mAh·g^−1^). However, the formation of harmful dendrites on its surface together with the unstable Li-electrolyte interface extremely reduces the widely commercial application of the metal lithium [18,19]. Other potential anode materials including, but not limited to, Li_4_Ti_5_O_12_ and Si are also faced with other troublesome obstructions: intrinsic low electrical conductivity and lithium diffusion co-efficient for spinel Li_4_Ti_5_O_12_, and for the Si anode, huge stresses coupled with drastic volume expansion during lithiation, and fracture and/or contact loss of the electroactive Si over cycling [20,21,22,23]. Hence, intensive research should be conducted to figure out the origin of structural evolution and to explore effective approaches to completely mitigate or eradicate the critical issues mentioned above.

Facing these challenges in LIBs, worldwide researchers devote themselves to seeking effective solutions, ranging from various nanostructured electrode materials, component optimization, novel cell configuration design, and so on [24,25,26,27,28]. Among these choices, surface coating of electrode materials already has been widely demonstrated to significantly improve the electrochemical properties of LIBs. Different types of coating materials, depending on the inherent feature of electrodes themselves, have been adopted and generalized [29,30,31,32]. Nevertheless, it is universally recognized that the adopted coating route plays a significant role in the ultimate performance of LIBs. Although traditional strategies, including mechanical mixing [9,31,33,34], sol-gel method [11,35,36,37] and hydrolysis-precipitation [19,29], have been developed greatly, these approaches cannot build an intact, homogeneous, steady yet thickness-controllable coating layer on the bulk electrode. Especially, the coating particles just disperse randomly on the surface by mechanical mixing method because no intermolecular forces get involved. Additionally, as for the sol-gel method, due to the heterogeneously distributed coating raw materials and the limited fluid dynamics, the thickness of the coating layer is greatly difficult to be uniform.

In conquering these upsetting issues, a promising strategy, called atomic layer deposition (ALD), has been an extensively researched hotspot, benefitting from its multifunctional capabilities and exceptional characteristics [38,39,40,41,42,43,44,45]. By virtue of the ALD technology, various elaborate nanostructured electrode materials including cathodes and anodes have been successfully fabricated and, more significantly, high aspect ratio conformity and high controllability of the thickness, composition and crystalline can be easily realized [46,47,48,49,50]. Consequently, the mass transfer diffusion and/or reaction kinetics, structural stability, and practical energy density of the ALD-fabricated materials will be substantially enhanced.

Stimulated by the ever-increasingly application of the smart ALD in LIBs, we summarized here the up-to-date progress and development of the ALD technique in advanced LIBs in view of the ever-expanding research. Following the introductory section, the working mechanisms and characteristics of the ALD are briefly presented. The elegant electrode-electrolyte sur-/interface engineering based on the ALD brings outstanding synergistic effects to the structural stability and electrochemical properties of electrode materials and solid-state electrolytes (SSEs). Thus, the latest progress and development of the ALD to optimize the sur-/interfacial surroundings of both cathodes and anodes are highlighted and specified in the third part. Finally, we outline the remarkable significance of the ALD in next-generation LIBs together with its potential utilizations in LIBs for future studies.

## 2. Brief Description of the ALD Method

**Figure 2:**
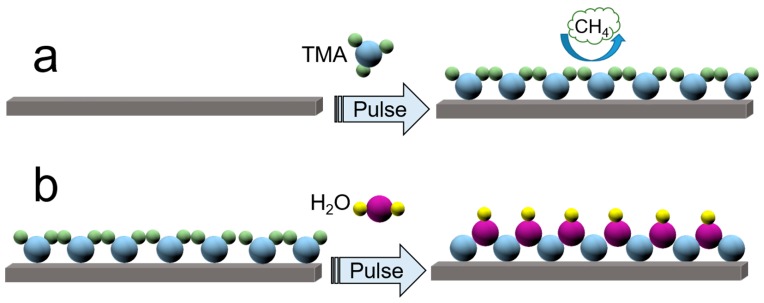
A model illustration for the atomic layer deposition (ALD) depositing Al_2_O_3_ on hydroxyl groups functionalized substrate including step (**a**) and step (**b**) by using the trimethylaluminum (TMA) and water as reactants.

The term “ALD” is originally dated from a technique called “atomic layer epitaxy” that was used to deposit ZnS for flat panel displays, and then greatly propelled under the background of booming semiconductor devices in consequence of the urgent requirement of continuous and pinhole-free films [43]. Following this, nowadays, appealing ALD has been widely recognized as a competitive approach to construct various types of materials and structures for many advanced and/or new studies and industry fields by virtue of its exceptional properties [38,51,52]. The surface chemistry, detailed mechanisms, and striking features including low growth temperature, atomic-scale and stoichiometric deposition, excellent uniformity and conformity peculiar to the ALD has been thoroughly summarized by Sun [41], George [53] and Zaera [54], et al. Multitudinous ALD-assisted applications have been presented in-depth by Puurunen [55], Gregorczyk [56] and co-workers. In general, the ALD goes on in a repeating manner, which depends on self-limiting, irreversible, and separated gas-solid surface reactions requiring typically at least two compounds. One should note that substrates, temperatures and precursors are the three crucial factors determining the final deposition features. Figure 2 illustrates the most representative yet successful ALD procedures for the ALD-depositing Al_2_O_3_ film by using trimethylaluminum (TMA) and water as precursors. In the first step (a), the precursor of the TMA reacts with the surface hydroxyl groups via self-limiting chemisorption to form a monolayer. The obtained surface is particularly reactive to the subsequent molecule, H_2_O, which forms a second monolayer in the following step (b). Thus, one cycle deposits one monolayer of the compound Al_2_O_3_ and regenerates the initial substrate with hydroxyl groups. By repeating the two reactions in sequence, the Al_2_O_3_ thin films can be grown with atomic precision and uniformity on planar, nonporous, or porous materials. More encouragingly, the ALD technique with high aspect ratio conformity and precise controllability of thickness, composition and crystalline is undoubtedly appealing for the preparation of nanostructured materials of rigorous geometry and composition, which only takes a few minutes. Furthermore, the ALD surface modification with no any additional detrimental reactant endows more uniform surface coverage than conventional wet chemistry methods. Thus, the ALD is deductively easy to scale up for industry-level applications in LIBs. Up to now, the ALD has been incessantly explored as an alternative approach to synthesize cathode and anode materials, as well as SSEs utilized in rechargeable energy applications, such as LIBs, lithium-sulfur batteries [57,58], sodium-ion batteries [59,60,61], lithium-O_2_ batteries [62], supercapacitors [63,64], solar cells [65], and so on. Especially in this review, we intensively focus on the up-to-date achievements of the ALD in synthesizing, modifying, stabilizing the electrodes and tailoring the electrode-electrolyte sur-/interfaces for high-performance LIBs. 

## 3. Construction of Electrode Materials and SSEs via the ALD Methodology

The contributions offered by nanostructured electrode materials can be summarized as follows: (i) enhanced cyclic stability; (ii) higher rate capability benefitted from the shorter diffusion path for Li^+^ ions and electrons; and (iii) less possibility of parasitic reactions with the bulk materials. Notably, the ALD has distinct advantages, such as thin-film thickness control at the atomic scale and excellent conformity, which are essential to realize electrode materials at nanoscale [66]. However, the nanostructured electrode still possesses some shortcomings, for instance, unsatisfactory processability caused by the large specific surface area (SSA), lower volumetric energy densities, and more complicated synthetic processes. Although nanomaterials synthesis and surface coating via the ALD have made a great amelioration for the aforementioned issues, the low ionic conductivity of conventional ALD coating materials is still an urgent problem to be solved. Fortunately, the SSEs can efficiently solve this challenge and conquer the dissolution puzzles [41,42]. Table 1 selectively summarizes the up-to-date anode and cathode materials, and SSEs constructed via the ALD.

### 3.1. Anodes via the ALD

Anodes synthesized via the ALD mainly focus upon the transition metal oxides (TMOs) including RuO_2_ [67], SnO_2_ [68,69,70,72,73,74], TiO_2_ [75,76,77,78,79,80,102], and ZnO [81]. Although these potential anode materials can yield high theoretical capacities (~781 mAh·g^−1^, corresponding to ~5400 Ah·L^−1^, greatly exceeding ~837 Ah·L^−1^ for the graphite), it is still confronted with many critical issues. For instance, the SnO_2_ is always obsessed by the exaggerated volumetric variation. In addition, the TiO_2_ anodes are so troubled by inherently low electronic and ionic conductivities [70,73,76]. As widely identified, carbonaceous materials with superior electronic conductivity and adjustable SSA, representative of graphene, CNTs and MXenes [66,103], are essentially demanded for high-rate capabilities. Therefore, the hybrid structures integrating carbon materials and TMOs are rationally constructed so as to take advantages of each constituent and fully exert their synergetic functionality. Due to the conformal and controllable features down to atomic scales, specific morphologies, structures, and electrochemical properties of the TMOs can be finely tailored by regulating ALD parameters including precursors, temperatures, and the surface functionality of carbon substrates [69,102].

**Figure 3:**
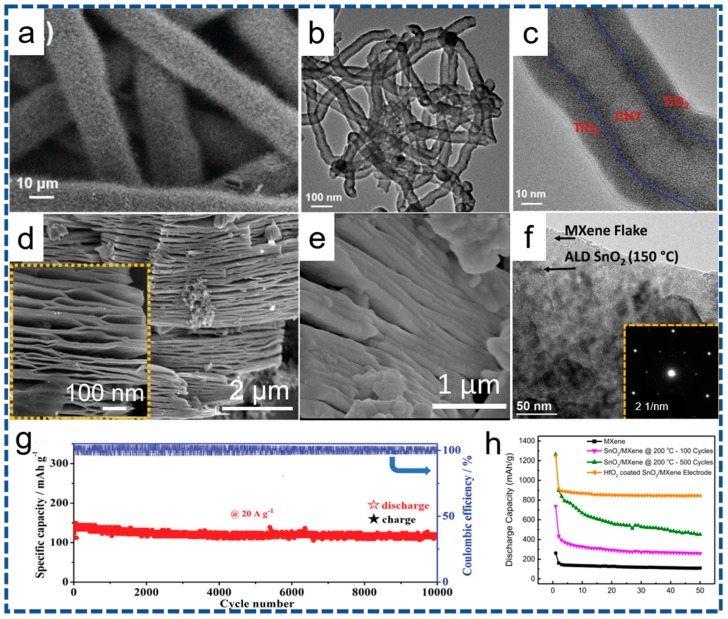
Typical anode materials fabricated via the ALD on carbonaceous supports. (**a**) Field emission scanning electron microscope (FESEM) and (**b**,**c**) Transmission electron microscope (TEM) images of TiO_2_@CNTs/CFP. Scanning electron microscope (SEM) images of (**d**) HF-etched Ti_3_C_2_ MXene and (**e**) SnO_2_-coated MXene anode, (**f**) TEM analysis of MXene sheets coated with a 50-nm-thick layer of SnO_2_ and selected area electron diffraction (SAED) pattern (the inset). (**g**) Cycling performance at 20 A g^−1^ for 10,000 cycles for TiO_2_@CNTs/CFP. (**h**) Cyclic performance over 50 cycles at 500 mA·g^−1^ of SnO_2_/MXene and HfO_2_ coated SnO_2_/MXene electrodes. (**a**–**c**,**g**) reprinted with permission from Ref. [75] @copyright 2016 Wiley. (**d**–**f**,**h**) reprinted with permission from Ref. [70] @copyright 2017 Elsevier.

A typical representative of TMOs-based anodes combining carbonaceous materials is amorphous TiO_2_ deposited on CNTs network/carbon fiber paper (CFP) substrates via the ALD at 120 °C, where the TiCl_4_ and water as the titanium and oxygen source, respectively [75]. The formed three-dimensional (3D) hierarchical TiO_2_@CNTs/CFP is shown in Figure 3a–c. Another analogous study was conducted by Fan et al. [104], where a thin TiO_2_ layer is deposited on the 3D hierarchical carbon nanowire (NW) array with mechanical robustness and flexibility. Apart from the 3D supports, Alshareef and co-workers recently reported the SnO_2_ grown on two-dimensional (2D) titanium carbide (MXene) sheets by the ALD, as exhibited in Figure 3d–f, and further utilized the SnO_2_/MXene composite anode for LIBs. These diversities in morphology and crystallinity can be attributed to be the variation in the surface functional groups and reaction mechanisms with different deposition parameters [105]. As expected, superior electrochemical performance is appealingly observed. As shown in Figure 3g, the outstanding Li-storage properties have been achieved for the amorphous TiO_2_@CNTs/CFP anode, exhibiting high reversible capacity (~272 mAh·g^−1^ at 0.1 A·g^−1^), superior rate capability (~133 mAh·g^−1^ at 40 A·g^−1^) and ultralong cycling performance (~93% capacity retention after 10,000 cycles at 20 A·g^−1^). Similarly, as shown in Figure 3h, the SnO_2_/MXene anode exploits a stable discharge capacity of ~843 mAh·g^−1^, and retains the mechanical and structural integrity of the 2D conductive MXenes platform meanwhile. The excellent Li-storage properties based on the 2D and/or 3D carbon materials can be put down to its structural uniqueness, which shortens the ionic diffusion length, buffers the volume changes, and supports large SSA for repeated Li^+^-insertion/extraction reactions.

Benefitting from these unique carbonaceous supports, several other types of 2D/3D nanostructures-based anodes fabricated by the ALD have been reported, such as, Li_4_Ti_5_O_12_@graphene nanosheets (GNs) [82], RuO_2_@MWCNT [67], TiO_2_@GNs [76], TiO_2_/carbon black [78], WS_2_@MWCNT [83], WN*_x_*@CNTs [85]. Apart from carbon-based matrixes, 3D nanoporous gold substrates was further applied for the TiO_2_ deposition [80], where large pore size is essential for Li^+^ diffusion, particularly at high rates. Additionally, electroactive SnO_2_ was conformally deposited on Ni nanofoam [68], where both large SSA and high conductivity are proved to contribute more electroactive sites for fast electrochemical reaction. As a result, the obtained SnO_2_/Ni nanofoam anodes show even higher capacities and better cycling ability. Furthermore, thin film anodes (MoN*_x_* [84] and LiTP [86]) directly prepared via the ALD are also significantly developed, and demonstrate highly stable structures and outstanding rate capability. Therefore, we have strong evidence to believe that the smart combination of the tunable thickness/mass of thin-layer electroactive phases and carbonaceous matrixes with superior electronic conductivity and large SSA has been shown very promising for high-performance energy storage devices.

### 3.2. Cathodes via the ALD

Owing to the exceptional reaction-controlled deposition process utilized by the ALD, it can deposit homogeneous and conformal thin films on substrates with high-aspect-ratio topography, and provide high flexibility for tuning the thickness, size, and composition of target materials at the atomic scale [48,106]. Thus, several unique yet beneficial characteristics of ultrathin materials grown by the ALD for electrochemical energy storage are anticipated to be observed. Actually, just several cathodes fabricated by the ALD have been investigated for LIBs so far, such as non-lithiated cathodes (V_2_O_5_ [87,88,89,90] and FePO_4_ [91]), and lithiated cathodes including LiFePO_4_ [48], LiCoO_2_ [92] and Li*_x_*Mn_2_O_4_ [93].

**Figure 4:**
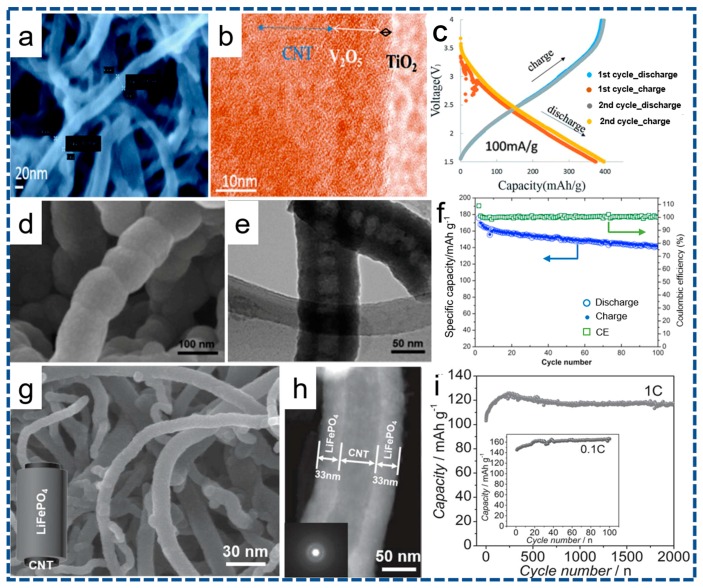
(**a**,**b**) SEM and TEM images of the TiO_2_/V_2_O_5_/CNTs paper electrode. (**c**) Voltage profile of the TiO_2_/V_2_O_5_/CNTs paper electrode for lithium ions storage. (**d**) SEM and (**e**) TEM image of the FePO_4_/NCNTs. (**f**) Cycling stability of the FePO_4_/NCNTs at a current density of 178 mA·g^−1^. (**g**) SEM and (**h**) TEM images of the LiFePO_4_@CNTs. (**i**) Cycling performance of the LiFePO_4_@CNTs cathode. (**a**–**c**) reprinted with permission from Ref. [90] @copyright 2016 Royal Society of Chemistry. (**d**–**f**) reprinted with permission from Ref. [91] @copyright 2015 Elsevier. (**g**–**i**) reprinted with permission from Ref. [48] @copyright 2014 Wiley.

**Figure 5:**
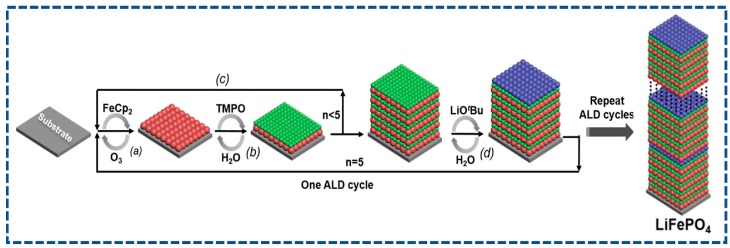
ALD synthesis of amorphous LiFePO_4_ at 300 °C using the FeCp_2_, O_3_, trimethylphosphate (TMPO), H_2_O, and lithium *t*-butoxide (LiO^t^Bu). (**a**) Sequential pulse of FeCp_2_ and O_3_ leading to the growth of a Fe_2_O_3_ layer (red); (**b**) sequential pulse of TMPO and H_2_O for deposition of a PO*_x_* layer (green); (**c**) steps (**a**,**b**) are repeated five times; (**d**) sequential pulse of LiO^t^Bu and H_2_O leading to formation of Li_2_O layer (blue). One ALD cycle for the growth of amorphous LiFePO_4_ consists of steps (**a**–**d**) reprinted with permission from Ref. [48].

V_2_O_5_, as the cathode material for LIBs, can be easily obtained via the ALD with no need for any thermal post-treatment. Lately, a 3D multi-hierarchical TiO_2_/V_2_O_5_/CNTs paper electrode has been investigated by Xie and co-workers [90], where amorphous V_2_O_5_ and ultrathin TiO_2_ are deposited on the CNTs paper by utilizing VOTP and H_2_O, and TiCl_4_ and H_2_O as precursors. Typical SEM and TEM images of the as-fabricated TiO_2_/V_2_O_5_/CNTs are displayed in Figure 4a,b. It can be observed that no agglomerated particles can be detected after ALD deposition, implying that TiO_2_ and V_2_O_5_ thin films are both uniformly anchored on the surface of CNTs. This distinctive TiO_2_/V_2_O_5_/CNTs electrode yields a discharge capacity of ~400 mAh·g^−1^ at 100 mA·g^−1^, as shown in Figure 4c, approaching the theoretical value of the V_2_O_5_. More significance of this work is that the dissolution problem of vanadium cathodes has been substantially resolved by the TiO_2_ coating. Another non-lithiated cathodes synthesized via the ALD is amorphous FePO_4_ [91]. Sun et al. develops a non-aqueous approach to deposit iron phosphate cathode materials on nitrogen-doped carbon nanotubes (NCNTs) by combining ALD subcycles of the Fe_2_O_3_ (ferrocene-ozone) and PO*_x_* (trimethyl phosphate-water) at temperatures between 200–350 °C [91]. Corresponding SEM, TEM images, and cyclic stability of the FePO_4_/NCNTs are depicted in Figure 4d–f, respectively. Evidently, the FePO_4_/NCNTs cathode delivers a discharge capacity of ~177 mAh·g^−1^ when evaluated as a cathode material for LIBs. The amorphous FePO_4_ fabricated by the ALD is expected to be applied in conventional LIBs and all solid-state thin film batteries as a surface-modification material. The most intriguing advancement in ALD cathodes is the synthesis of LiFePO_4_ on conductive CNTs as a high-performance cathode for LIBs, recently reported by Sun et al. [48]. TEM images of the LiFePO_4_/CNTs are shown in Figure 4g,h, which confirms the uniformity of the as-deposited LiFePO_4_ layer with thickness of about 33 nm on the CNTs. The corresponding electrochemical properties are provided in Figure 4i. Furthermore, the conforming representative ALD sequence of 5 × (FeCp_2_-O_3_-TMPO-H_2_O) + 1 × (LiO^t^Bu-H_2_O) is exhibited in Figure 5. Impressively, a high capacity of ~71 mAh·g^−1^ can be delivered by the LiFePO_4_/CNTs electrode as the current rate increases up to 60 °C. In addition, this LiFePO_4_/CNTs cathode owns superior capacity retention of ~70.5% after 2000 cycles. The smart combination of carbon nanostructure and ultrathin ALD layer can be responsible for the excellent rate and cycling ability. More meaningfully, this work neatly paves the way towards purposeful fabrication of olivine LiMPO_4_ cathodes as a high-power cathode for next-generation LIBs.

The other two lithiated cathode materials, namely layered LiCoO_2_ [92] and spinel LiMn_2_O_4_ [93], which are widely applied in commercial electrical devices, also has been successfully deposited by combining a Li_2_O subcycle with another one or two binary oxide ALD subcycles. In order to further explore how to achieve the miniaturization of LIBs, Notten et al. [92] rationally adopts a remote plasma ALD process to fabricate LiCoO_2_ cathode materials from ALD subcycles of Co_3_O_4_ (CoCp_2_-plasma O_2_) and Li_2_CO_3_ (LiO^t^Bu-plasma O_2_) in a remote plasma ALD system performed at 325 °C, which is followed by calcination at 700 °C for 6 min. Corresponding investigations manifest that the ALD-deposited LiCoO_2_ thin film electrodes (~60% electroactive material) show good reversible electrochemical performance. Another significant study is the spinel Li*_x_*Mn_2_O_4_ cathode, which is obtained from (Mn(thd)_3_-O_3_) and (Li(thd)-O_3_) subcycles at a deposition temperature of 225 °C [93]. Particularly, it is worth mentioning that this contribution provides an attractive route to transform ALD-MnO_2_ and ALD-V_2_O_5_ into Li*_x_*Mn_2_O_4_ and Li*_x_*V_2_O_5_ by Li(thd)/ozone and LiO^t^Bu/water treatments, respectively. As a result, the Li*_x_*Mn_2_O_4_ fabricated via the ALD exhibits ~230 mAh·g^−1^ at 50 μA and stable cycling performance over 1000 cycles. Remarkably, these significant results powerfully reveal that the ALD is a promising method to deposit electroactive lithium-containing electrode materials for advanced LIBs.

### 3.3. SSEs via the ALD

Recently, all-solid-state LIBs are becoming hot research topics, thanks to their enhanced safety and pronounced cyclic stability. However, all-solid-state batteries are still confronted with two critical challenges including high interface resistance of the electrodes and poor ion conductivity of the SSEs themselves [107,108]. Benefitting from its superior capability to conformally coat the electrode surface, the ALD has been served as an alternative, promising candidate to fabricate all-solid-state thin film batteries [109]. Several papers previously reported have demonstrated the meaningful contribution of the ALD in this domain, especially solid electrolyte film functioning as a protective layer [67,100,110,111,112,113,114]. In fact, some SSEs prepared by the ALD have been investigated in recent studies, as summarized in Table 1, such as LiPON [94,95], Li_7_La_3_Zr_2_O_12_ [96], Li*_x_*Al*_y_*Si*_z_*O [97,98], Li*_x_*Ta*_y_*O*_z_* [99], Li*_x_*Al*_y_*S [100] and Li_2_O-SiO_2_ [101]. These lithiated SSEs provide a potential platform for achieving high energy/power density, long-lasting, and safe rechargeable batteries. Typically, the ceramic oxide garnet Li_7_La_3_Zr_2_O_12_ (LLZO) with Al-doped reported by Dasgupta [96] for the first time is deposited at 200 °C from Li_2_O (LiO^t^Bu-H_2_O), La_2_O_3_ (LaFAMD-H_2_O), ZrO_2_ (TDMAZ-H_2_O), and Al_2_O_3_ (TMA-H_2_O) with a series of subcycles of La(FAMD) + O_3_, TDMAZ + O_3_, or LiO^t^Bu + O_3_. Intriguingly, this cubic LLZO SSE successfully applied to the solid-state Li-S batteries has been certified to yield an attractive specific capacity either at room temperature (RT) or at high temperatures, as well as high Coulombic efficiency (CE) and remarkable electrochemical stability [107]. Lithium silicates as another promising solid state thin films are deposited at temperatures between 225 and 300 °C by combining ALD Li_2_O and SiO_2_ subcycles using LiO^t^Bu, TEOS, and H_2_O as precursors [101]. The investigation shows that the lithium silicate thin films can exhibit an ionic conductivity of 1.45 × 10^−6^ S·cm^−1^ at 373 K due to the higher lithium concentration and lower activation energy. Therefore, with the unique superiorities to substantially decrease the internal resistance and overall battery impedance, the ALD is anticipated to exert a tremendous boost in fabricate ultrathin and functional SSEs for all-solid-state micro-batteries.

## 4. Sur-/Interfacial Engineering Optimization via the ALD

In general, the interfacial side-reactions always unavoidably emerge between the electrode and liquid electrolyte during repetitive charge-discharge process [10]. On the one hand, the tunability and controllability of the sur-/interface reactions will be tremendously beneficial to enhance the electrochemical performance of LIBs. For instance, as for the anodes, the solid electrolyte interphase (SEI) at its interface is critical to protect the bulk material from destruction and improve thermostability by preventing adverse decomposition of the LiFP_6_-based electrolytes containing trace water [13,32]. On the other hand, the interface side effects derived from the direct contact of electroactive material and electrolytes also can lead to a variety of unfavorable influences, particularly increasingly accumulated irretrievable damage to the cathodes, which intensely compromises the overall properties of LIBs. Moreover, these detrimental effects will be aggravated under the rigorous high cut-off voltages and high temperatures. Typically, the capacity loss in the initial discharge process mainly results from the SEI formed on the anode surface, which resulting in permanent consumption of Li^+^ ions. As for the cathode surface, the byproducts (Li*_x_*POF*_y_*, P_2_O_5_, LiF, etc.) generated from the side reactions tightly adhere to the cathode surface, which highly retards the Li^+^ transfer [10,33,115]. Of note, the gradually enlarged phase distortion and the dissolution of active elements are also accompanied meanwhile, which subsequently renders a sharp decline in electrochemical Li-storage performance.

**Figure 6:**
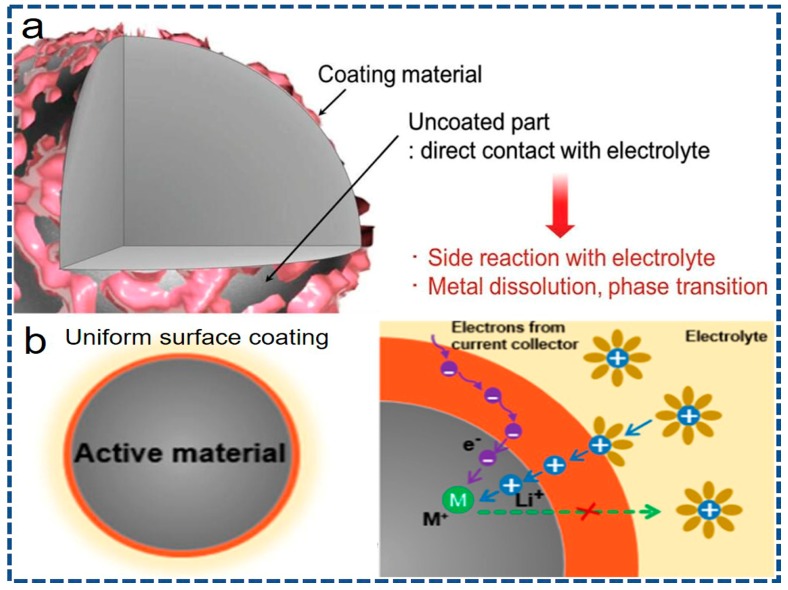
(**a**) Typical schematic views of coated surface morphologies prepared from conventional method with an uncoated part and an island-shape coating material (reprinted with permission from Ref. [116] @copyright 2014 Wiley). (**b**) Schematic diagram of an ideal surface coating layer on the electroactive materials (reprinted with permission from Ref. [40] @copyright 2015 IOP).

In order to substantially improve the interfacial mass transportation, surface coating strategy has been widely verified to effectively address the critical issues encountered by most of the electrode materials including phase deformation, lithium-containing impurities accumulation and electroactive elements dissolution. Importantly, it is recognized that the thickness, uniformity, conductivity and stability of the coating layer is still full of challenges. As obviously shown in Figure 6a, conventional and/or improper coating routes are readily inclined to yield intermittent and scattered coating products on the host material. The exposed cathode/anode surface is unprotected when faced with the electrolytes, resulting in the poor electrochemical properties [116]. As vividly depicted in Figure 6b, constructing the desirable surface coating layer should at least satisfy the following requirements: (I) ultrathin, intact and consecutive coating layer. Ultrathin coating layer is not only conducive to the transmission of ions and electrons, but also has a positive impact on the capacity of the bulk electrode. The intact and consecutive coating layer will maximize the preservation of bulk materials and guarantee the smooth diffusion of Li^+^ ions and electrons through the coating layer. (II) High conductivity for Li^+^ ions and/or electrons. The coating layer of high conductivity can effectively decrease the charge transfer resistance and electrochemical polarization, thus facilitating the migration of Li^+^ ions or electrons into/out of the bulk material. (III) Superior chemical durability. The coating layer should have superior chemical durability so as to keep off the corrosion from electrolytes, assuring the repeated charge/discharge cycles [7,10,40]. Therefore, the exploration for an undefective coating approach is always in progress.

The ALD is identified to be an extraordinary deposition technique combining atomic scale deposition performed at low operating temperatures and outstanding controllability in the thickness and dimensions of the coating layer. The ALD surface engineering on the anodes and cathodes are summarized in Table 2 and Table 3, respectively. It can be easily concluded that both of structural stability and electrochemical properties of anodes and cathodes are significantly enhanced after ALD coating. Furthermore, especially in LIBs, the ALD has been utilized in various functionalities including non-conductive (mainly refer to the TMOs) and conductive (lithium-containing SSEs) surface coating for anode and cathode materials. As retrieved, the ALD coating is performed: (I) on the anodes and cathodes with one or more target materials [117,118,119]; (II) on the electrodes in order to protect an electronically conductive network among the electroactive materials, carbon additives, and current collectors [120,121,122]; (III) directly on the as-prepared powders before assembling cells [123,124,125,126,127]. More importantly, the whole ALD process is energy-efficient, fast (only a few minutes), no any damage to the electrodes, thus stands a chance in practical industrial applications. As a consequence, the remarkable functionality of the ALD surface coating and consequential contribution to enhance the electrochemical properties will be highlighted in this section.

### 4.1. Sur-/Interfacial Engineering in Anodes via the ALD

The ALD sur-/interfacial engineering in the anodes mainly aims to address a hard nut to crack stemming from the SEI. The formation mechanisms on anodes and the intrinsic nature of the SEI has been evaluated in-depth for LIBs [127,128]. The universally accepted conclusion is that the special compositions and structures are both critical factors in determining electrochemical performance and degeneration of LIBs. Especially for the Si, Sn and SiO_2_ anodes, the demotivated influences derived from SEI formation aggravate intensely during the cyclic process along with large volume variation, which can damage the anodes and thus expose the fresh surface, resulting in the great loss in capacities [123,129,130]. 

As a realistic anode candidate for commercial LIBs up to now, the graphite provides a combination of high capacity, low potential and moderate volume change [7]. However, the SEI formed on the surface starting from the first cycle gives rise to potential safety issues, particularly at high temperatures. To preserve the original surface of the graphite, the ALD has been carried out to enhance the cyclic stability and safety at elevated temperatures. Lee’s group [121] directly deposits a conformal ultrathin Al_2_O_3_ on a graphite electrode rather than the powders. The unique Al_2_O_3_ film, as a protective coating shell, does not disrupt inter-particle electronic pathway at all, which consequently contributes to considerable enhancement in both long-term durability and safety. Corresponding electrochemical evaluation shows that the graphite anode with 5 Al_2_O_3_ ALD cycles retains ~98% of the initial capacity after 200 charge/discharge processes at 50 °C, while the capacity of the bare counterpart dramatically fades to no more than ~26% at the same cases. Another intriguing work [122] deducted that the TiO_2_ surface modification functions as an artificial SEI to ameliorate the stability and capability of graphite electrode under the high-temperature operation.

**Figure 7:**
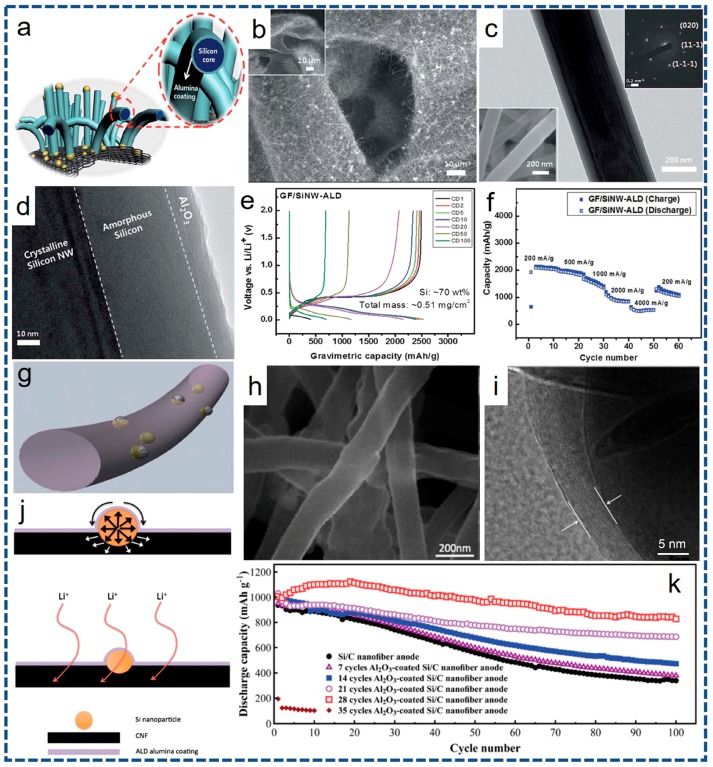
(**a**) Schematic illustration of ALD Al_2_O_3_-coated Si NWs grown on 3D porous graphene foam. (**b**) SEM images of Si NWs. (**c**) TEM and (**d**) High resolution transmission electron microscopy (HRTEM) images of a single Al_2_O_3_-coated Si NW. (**e**) Voltage profiles of for the 1st, 2nd, 5th, 10th, 20th and 50th cycles of Al_2_O_3_-coated Si NWs. (**f**) Rate capability of the Al_2_O_3_-coated Si NW composite with various currents as indicated. (**g**) Schematic of Al_2_O_3_-coated Si/C composite NFs. (**h**) SEM and (**i**) TEM image of Al_2_O_3_-coated Si/C composite NFs with 28 ALD cycle numbers. (**j**) Schematic of physical/mechanical (upper) and chemical protective effect (down). (**k**) Cycling performance of Si/C composite NFs and Al_2_O_3_-coated Si/C composite NFs. (**a**–**f**) reprinted with permission from Ref. [129] @copyright 2016 Royal Society of Chemistry. (**g**–**k**) reprinted with permission from Ref. [132] @copyright 2014 Royal Society of Chemistry.

It has been widely proved that replacing low-capacity carbon anodes with the Si can tremendously improve the energy-storage capacity of LIBs. Unfortunately, the practical utilization of Si anode remains hindered by several issues. The trickiest part is the drastic volume changes during lithiation/delithiation processes, rendering electrical disconnection and capacity dropping [131]. A desirable surface coating has thus been pursued to settle this knot. As reported by Güneş, silicon NWs have been successfully synthesized on a 3D porous graphene foam combining with an ALD Al_2_O_3_ film of ~10 nm in thickness, as depicted in Figure 7a. Corresponding SEM and TEM images are shown in Figure 7b–d. Taking this unique ALD-assisted Si composites as anode material in LIBs, it yields high gravimetric and areal capacities, as well as comparable cycle life. As provided in Figure 7e,f, after 20 charge/discharge cycles, ALD Al_2_O_3_-coated Si NWs composites shows ~83% capacity retention while the uncoated sample retains only ~76% of the first cycle. At the 50th cycle, the discharge capacities were decreased to ~1125 and ~875 mAh·g^−1^ for the ALD-assisted and bare samples, respectively. An ALD Al_2_O_3_ coating layer on Si NWs not only cuts down the byproducts from side reactions but also reinforces the mechanical stress during the electrochemical reaction [49].

Zhang and co-workers have thoroughly investigated the effects of ALD Al_2_O_3_ coatings with different thicknesses on electrochemical performance of Si/C composite nanofibers (NFs) [132]. As shown in Figure 7g, Si/C composite NFs are obtained via electrospinning and following carbonization. The thickness of the subsequent ALD Al_2_O_3_ coating layer is tuned at the monolayer-scale via adjusting the number of ALD cycles. Corresponding SEM and TEM images of Al_2_O_3_-coated Si/C composite NFs with 28 ALD cycle numbers, as represented in Figure 7h,i, indicate that both the NFs and exposed Si nanoparticles are coated with bright shells corresponding to the Al_2_O_3_ coatings with a thickness of ~5 nm. This study powerfully proves that the ALD Al_2_O_3_ coating can favor for maintaining the structure integrity and mitigating capacity fading, and further acts as an artificial SEI film to stabilize the electrode surface and to prevent undesirable side reactions during cycling, as vividly depicted in Figure 7j. The capacity retention at 100th cycle, as plotted in Figure 7k, increases significantly from 36.1 to 82.3% and corresponding CE increases from 98.4 to 99.9%, compared to those of the uncoated Si/C NFs. Another positive case by Lu and co-workers [130] validates in detail that an ALD TiO_2_ buffering layer (~3 nm) remarkably affords the Si volume change inside the spheres during repeatedly alloying and de-alloying, which largely suppresses the pulverization and aggregation of Si particles. As a result, the core-shell Si@TiO_2_ anode material fabricated by ALD delivers a superior CE and charge capacity of ~98% and ~1580.3 mAh·g^−1^ after 50 cycles. In addition, further studies [123,124,125] also confirm the cyclic stability of the Si anodes can be substantially enhanced after ALD surface coating. 

**Figure 8:**
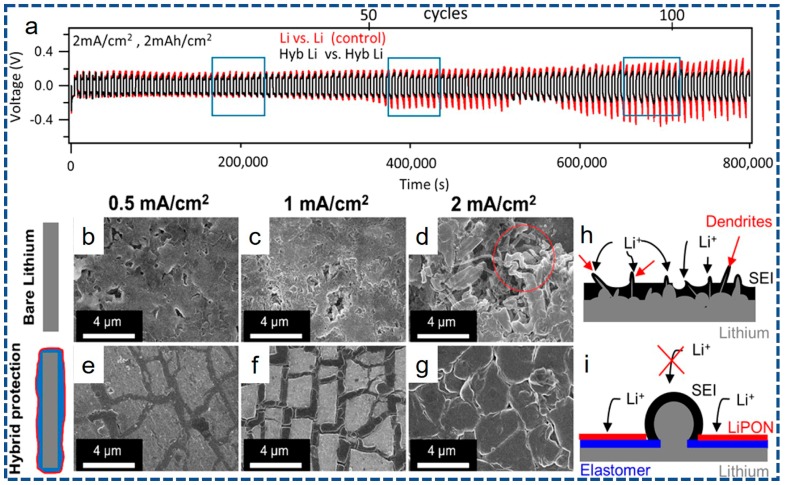
(**a**) Voltage profiles of bare Li/Li (red) and hybrid protected Li/Li at a high current density of 2 mA·cm^−2^ and a capacity of 2 mAh·cm^−2^. (**b**–**d**) SEM images of bare Li metal and (**e**–**g**) hybrid-protected Li metal cycled for 100 cycles with varied current densities. Mechanistic schematics for (**h**) the bare Li and (**i**) hybrid elastomer/LiPON protection showing Li metal surface layer evolution after 100 cycles at current densities of 2 mA·cm^−2^. (**a**–**i**) reprinted with permission from Ref. [127] @copyright 2017 American Chemical Society.

The Li metal is regarded as the most attractive anode materials for LIBs, however, indisciplinable lithium dendrites growth leads to inferior cycling efficiency and severe safety issues, partially dragging the Li anode out of realistic applications [133]. In order to inhibit the detrimental reduction of electrolyte species on the Li metal surface, recently, Noked’s group [127] prospectively develop a novel approach to stabilize Li metal surface by rationally employing unique ALD together with self-healing electrochemical polymerization (EP). Consequently, a hybrid organic/inorganic artificial solid electrolyte interphase (ASEI) including organic DOL and a thin inorganic ALD LiPON layer (~15 nm), uniformly deposits upon the Li metal surface. Verification by electrochemical properties at increased current density and corresponding capacity is shown in Figure 8a. The corresponding blue rectangle from left to right stands for early cycle life, cycle life at Li/Li cells showed unstable profiles (Li anode degraded), and cycle life at Li/Li cells’ failure, respectively. Apparently, early degeneration starts from only 55 cycles for bare Li cells, and the obvious failure occurs after 80 cycles. Nevertheless, the symmetric hybrid protected cells demonstrate stable cycling behavior up to over 110 cycles. Besides, SEM images for the surface changes of both electrodes ending on a Li plating cycle after 100 cycles, as represented in Figure 8b–g, further validate the superiorities of the ALD in controllability and uniformly coating. Clearly, Li dendrites distribute randomly on the surface of the unprotected Li, as a sharp contrast, the Li metal anode after ALD coating displays a stable, robust surface morphology, as distinctly depicted in Figure 8h,i. Accordingly, the coalition of an organic layer and an inorganic layer by the ALD is found to synergistically accommodate morphological and mechanical changes related to Li insertion/de-insertion. Equally, ultrathin Al_2_O_3_ protective layer [134,135] and Li*_x_*Al*_y_*S SSE [100] are of date employed to stabilize the electrode surface via the ALD. These in-depth investigations further demonstrate the unique advantages for sur-/interfacial modification via controllable and uniform ALD coating layer. Therefore, we have well-founded reasons to believe that the critical puzzles confronted by the Li metal anode can be considerably ameliorated via the advanced ALD technique.

Other conversional and intercalation metal oxides, sulfides, and Li-inserted compounds also obtain markedly improved structural and electrochemical properties, such as SnS_2_ [136,137], SnO_2_ [138], MoO_3_ [139], Fe_3_O_4_ [140], Fe_2_O_3_ [141], CuO [142] and Li_4_Ti_5_O_12_ [120]. As demonstrated by Alshareef et al., an optimal HfO_2_ layer (~1 nm) corresponding to 10 ALD cycles effectively compromises between Li^+^ ion diffusion and passivation effects. The exploration shows that ALD HfO_2_-coated MoO_3_ nanorods (NRs) tend to stabilize faster than the bare one due to the HfO_2_ coating layer [139]. Sun’s group has studied and testified the accelerative function contributed by an ultrathin ~2 nm ALD ZrO_2_ surface coating shell on the Li_4_Ti_5_O_12_ anode. Evidently, the ZrO_2_ coating layer can enormously suppress SEI formation and effectively facilitate the Li^+^ ions diffusion through SEI film, and thus expand the potential window of Li_4_Ti_5_O_12_ anode for higher energy density [120].

### 4.2. Sur-/Interfacial Engineering in Cathodes via the ALD

As extensively proved, the undesirable detrimental side-reactions are readily triggered off when the surface of cathode materials is directly exposed to the liquid electrolytes, including the electrolyte decomposition, irreversible phase degradation, and the oxygen loss and transition metals dissolution starting from the surface region [10,164]. Thus, rational surface coating depending on the surface characteristics of cathodes themselves has been widely implemented to restrict to the maximum the occurrence of side-reactions. Various compounds including metal oxides, phosphates and fluorides are utilized and studied for the surface coating. The coating layer can be functioned as a physical isolated film or HF scavenger, thus outstanding cyclic stability and rate capability are achieved. As for coating approaches, the ALD, in sharp comparison with the conventional mechanical mixing and sol-gel methods, affords extraordinary homogeneous cladding layer on the surface with precisely regulated down to sub-nanometers levels [5,164]. In this part, we comprehensively summarize the surface engineering via the ALD for cathode materials including layered cathodes, such as LiCoO_2_ [111,112,118,148,149], LiNi*_x_*Mn*_y_*Co*_z_*O_2_ [113,114,119,154,155,156,157], and Li-rich *x*Li_2_MnO_3_·(1 − *x*)LiMO_2_(M = Mn, Ni, Co) [161,162,163], and spinel cathodes, such as LiMn_2_O_4_ [150,151,152,153] and LiNi_0.5_Mn_1.5_O_4_ [52,158,159,160], as listed in Table 3. After careful observation in Table 3, we can discover that the compounds serving as ALD coating layer for cathode materials can be mainly divided into four categories: metal oxides (Al_2_O_3_ [118,119,152,154,155,156,159,161,163,165,166], TiO_2_ [118,153,159,161,166], ZrO_2_ [118,150], MgO [157], CeO_2_ [151], and Ga_2_O_3_ [119]), fluorides (AlF_3_ [148] and AlW*_x_*F*_y_* [111,112]), and phosphates (AlPO_4_ [160,162] and FePO_4_ [158]), and lithium containing compounds (LiAlO_2_ [52], LiTaO_3_ [114] and LiAlF_4_ [113]). Subsequently, according to the different function mechanisms based on these four types of coating materials, the internal relationship between the textures of constructed ALD cathode materials and their superior electrochemical properties is elaborately reviewed here.

**Figure 9:**
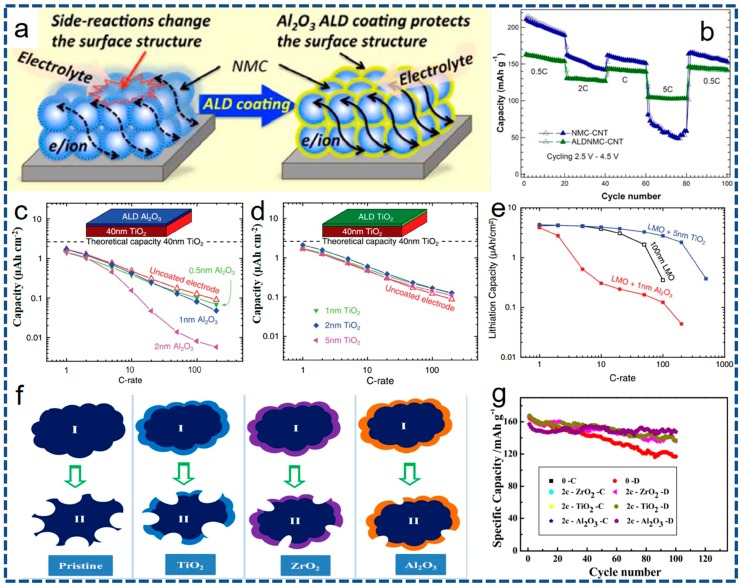
(**a**) Schematic illustration of comparison for bare and Al_2_O_3_-coated NMC electrodes. (**b**) High-voltage cycling performance of the bare and Al_2_O_3_-coated NMC electrodes cycled at 2.5–4.5 V (vs. Li/Li^+^). (**c**,**d**) De-lithiation capacity of the electrodes measured by charging and discharging the films at current densities varying from 1 to 200 °C between 1.0 and 3.0 V (vs. Li/Li^+^). (**e**) De-lithiation capacity of the bare and ALD-coated LiMn_2_O_4_ electrodes measured by charging and discharging the films at current densities at 3.5–4.5 V (vs. Li/Li^+^). (**f**) Schematic comparison of Co dissolution of LiCoO_2_ with various coating layers. (**g**) Comparison in cycling performance of LiCoO_2_ electrodes coated by different TMOs. (**a**,**b**) reprinted with permission from Ref. [155] @copyright 2015 American Chemical Society. (**c**–**e**) reprinted with permission from Ref. [153] @copyright 2017 Wiley. (**f**,**g**) reprinted with permission from Ref. [118] @copyright 2014 Elsevier.

Similar to the studies of employing ALD to optimize anodes, metal oxides ALD coating (particularly Al_2_O_3_) are the first applied and most concerned for cathode material surface modification. Up to now, the Al_2_O_3_ coating has been widely utilized to stabilize the surface structure. Typically, as earlier investigation by Scott’s group [147] show, electrochemical properties of the LiCoO_2_ are substantially enhanced with the Al_2_O_3_ coating layer deposited with a thickness of ~1–2 nm. The results show that the ALD Al_2_O_3_-coated electrodes have no capacity loss after 200 charge/discharge cycles at 2.8 C, in sharp contrast with the pristine one. In addition, the high-rate capability is also largely improved, which can be ascribed to the effective conservation of the electrode structure. Following this wave, other materials, such as LiMn_2_O_4_, LiNi_0.5_Mn_1.5_O_4_, and a series of Ni-based cathode including NMC333, NMC442 and NMC532, as well as Li-rich layered materials (such as Li_1.2_Ni_0.13_Mn_0.54_Co_0.13_O_2_) are well developed. Further convincing evidence for the positive role of the Al_2_O_3_ coating via the ALD on LiNi_0.4_Mn_0.4_Co_0.2_O_2_ has been recently probed by Ban and co-workers [155]. The Al_2_O_3_ layer mitigates the detrimental side reactions under high cut-off voltages (>4.4 V), without restricting the uptake and release of Li^+^ ions, as conveyed in Figure 9a. This protective layer endows more highly active Ni oxide sites, thus promoting charge compensation via the oxidation of Ni and deserving high-rate capability, as shown in Figure 9b. Although the ALD Al_2_O_3_ coating layer, acting as multifunctional roles in preserving the surface structural and chemical durability, the electronic conductivity of the working electrode will decline along with the increase in coating thickness [161,163]. Thus, the thickness of the coating layer should be precisely controlled via the ALD so as to guarantee the high electronic transportation. In consequence, other TMOs, representative of TiO_2_ and ZrO_2_, have been persistently pursued as coating layer by the ALD. Recently, the comparison of ultrathin ALD Al_2_O_3_ and TiO_2_ integrating with spinel LiMn_2_O_4_ is detailed conducted by Mattelaer and co-workers [153]. Apparently, it can be observed from Figure 9c,d that an Al_2_O_3_ coating layer as thin as 0.5 nm already leads to a dramatic slow-down of the electrode kinetics. In addition, the Al_2_O_3_ of ~1 nm in thickness cuts down the 200 °C capacity to half as compared to the uncoated TiO_2_ electrode, and with a ~2 nm ALD Al_2_O_3_ only 7% retains. In stark contrast, the buried TiO_2_ even for the ~5 nm coating still makes up more than 85% of the electrode. As reflected from the rate behaviors (Figure 9e), the rate capability is unaffected by the TiO_2_ coating since it does not pose an additional impedance on the electrode, while the ALD Al_2_O_3_ coating is obviously detrimental due to the 1.5 × 10^12^ Ω·cm resistivity toward Li^+^ ions. A significant study for the metal oxides ALD coating, Sun’s group simultaneously studies and compares the specific influences of ALD Al_2_O_3_, TiO_2_ and ZrO_2_ on LiCoO_2_ powders (I) vs. electrodes (II) and cycling and rate properties [118], as shown in Figure 9f and g, respectively. Apparently, electrochemical results fully confirm that the Al_2_O_3_ ALD coating affords the best cyclic stability while the ZrO_2_ ALD coating contributes to the best rate capability, resulting from the different intrinsic electronic conductivities of the two. This work also demonstrates that excessive ALD cycles will lead to inferior rate capability regardless of any metal oxide. Apart from the metal oxides mentioned above, other metal oxides such as CeO_2_, MgO and Ga_2_O_3_ are also successfully utilized via the ALD for surface modification of cathode materials. Interestingly, studies from Liang’s group testify that a pin-hole free ALD layer of CeO_2_ modified on LiMn_2_O_4_ electrode can not only protect the electrode from harmful side reactions but also function as an electronic transportation path [151]. Therefore, we can ascertain that the metal oxide coating is a competitive technique to improve electrochemical properties of the cathodes, and the metal oxides should not be confined to the traditional metal oxides (e.g., Al_2_O_3_). Several critical factors including the coating thickness, electrical conductivity and electrical band structure, as well as the fracture toughness of the ALD metal oxides should be simultaneously taken into account [38,42]. 

**Figure 10:**
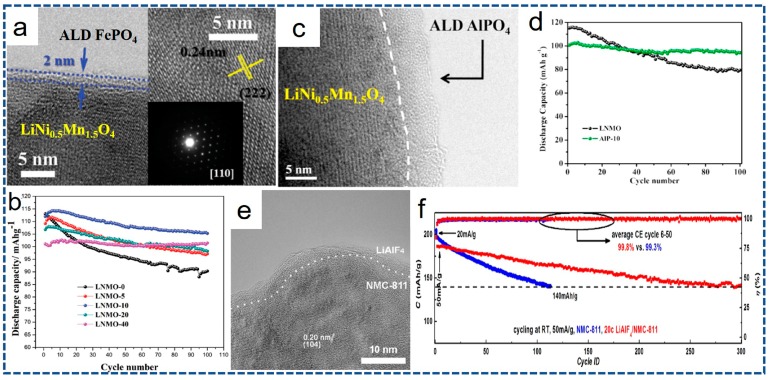
(**a**) HRTEM images and (**b**) cyclic stability of the ALD FePO_4_-coated LiNi_0.5_Mn_1.5_O_4_ cathode. (**c**) HRTEM image and (**d**) cycling stability of the AlPO_4_-coated LiNi_0.5_Mn_1.5_O_4_ cathode. (**e**) TEM image of the LiAlF_4_-coated NMC811 electrode after cycling. (**f**) Cycling behaviors of the pristine and LiAlF_4_-coated NMC811 electrodes at room temperature (RT) within an electrochemical window of 2.75–4.50 V (vs. Li/Li^+^). (**a**,**b**) reprinted with permission from Ref. [158] @copyright 2015 Wiley. (**c**,**d**) reprinted with permission from Ref. [160] @copyright 2017 Elsevier. (**e**,**f**) reprinted with permission from Ref. [113] @copyright 2017 American Chemical Society.

Recently, Sun’s group innovatively pioneered the ALD surface modification by using phosphates including FePO_4_ and AlPO_4_, which are directly deposited onto the initial powders rather than the whole electrode [158,160,162]. These phosphates with highly electronegativity of PO_4_^3−^ with Al^3+^/Fe^3+^ ions and amorphous structure do not trigger off any lattice stress, and consequently offer unhindered tunnels for Li^+^ ions and considerable electronic conductivity. The HRTEM images displayed in Figure 10a reveal that the ultrathin ALD FePO_4_ surface coating can be controlled to ~2 nm in thickness. From electrochemical evaluation in Figure 10b, the ALD FePO_4_-coated samples evidently yield mounting capacity retention with increasing ALD cycles, which powerfully certifies the protective feature of the ALD FePO_4_ shell. This can be put down to the significant suppression of Jahn-Teller distortion by ALD FePO_4_ layer. Thus, the formation and dissolution of Mn^2+^ in the byproduct of HF decrease accordingly [158]. The equal study of great significance is that AlPO_4_ successfully serves as protective layer by the ALD, outstanding electrochemical properties are plotted in Figure 10c,d. The well-proportioned ultrathin AlPO_4_ layer not only functions as a protective layer to insulate active material and electrolyte, but also evolves into an artificial SEI layer upon cycling, substantially inhibiting the decomposition of electrolytes and integrating the surface structure [160]. Therefore, in comparison with the most widely studied of metal oxides, phosphates-based surface modification provides an alternative option, and has great opportunities to improve other energy storage systems according to this design. 

An innovative promotion for ALD surface coating is to exploit some new and reasonable coating materials with desirable properties. Recently, in view of the insulating characteristics of metal oxides and relatively lower electrical conductivity of phosphates, lithium-containing compounds, as an ALD surface coating phase, has been investigated. For example, the possibility of building ultrathin ion-conducting LiAlO_2_ layers on LiNi_0.5_Mn_1.5_O_4_ electrode was initially explored by Park and coworkers [52]. After comparison with conventional ALD Al_2_O_3_ layers, the LiAlO_2_-coated electrode exhibits much more improved cyclic stability and rate capability, resulting from the better ionic conductivity of LiAlO_2_ than that of the Al_2_O_3_. In another representative study, the LiTaO_3_ SSE, as a ALD coating material, with a high ionic conductivity of 2 × 10^−8^ s·cm^−1^ at room temperature (RT) is represented by Li and co-authors [114]. As a result, the surface structure degradation is remarkably decreased even employing a high cut-off voltage 3.0–4.8 V and the ALD LiTaO_3_ coated electrode displays extraordinary electrochemical properties even at a high temperature of 55 °C. Lately, in order to operate at a wide electrochemical window to maximize energy density of the NMC811, Cui’s group [113] has been successfully developed a LiAlF_4_ solid thin film with robust stability and satisfactory ion conductivity as a coating layer via the ALD. Typical TEM characterization of 50 ALD cycles of LiAlF_4_-coated NMC811 electrode after cycling is shown in Figure 10e. Clearly, the sturdy LiAlF_4_ layer is still completely unscathed and uniformly distributed on the surface of NMC811 particles after cycling. As reflected in Figure 10f, the electrode with the LiAlF_4_ coating shell retains a capacity higher than ~140 mAh·g^−1^ after 300 cycles, while for the pristine one, the capacities decline sharply to <140 mAh·g^−1^ after 113 cycles. This can be reasonably attributed to the Li^+^-conductive fluoride-based interfacial layer fabricated by the ALD, which is thermodynamically stable within a wide electrochemical window. As a result, the stability of the high-performance NMC811 electrodes is greatly enhanced.

## 5. Concluding Remarks and Outlooks

Nowadays, LIBs are the most widely applied and competitive energy-storage devices. However, they are still confronted with a series of critical issues, which definitely results from the deviant evolution process of electrode materials, regardless of cathodes or anodes. The pursuit of desirable electrode materials with remarkable physicochemical properties is ongoing. Over recent decades, the ALD, benefitting from its atomic accuracy and uniformity, has been rapidly advanced as a promising approach to construct high-performance electrode materials and SSEs for predesigned objectives, which are commonly difficult or even impossible to achieve by other common techniques. More significantly, the ALD also has been fully demonstrated to be a quite accurate and efficient method for electrode sur-/interface modifications. Hence, the ALD embodies several key advantages over conventional methodologies in coping with tough challenges associated with LIBs. 

In this context, the up-to-date advances of the ALD in high-performance LIBs for designing and constructing prospective electrode materials and SSEs are comprehensively reviewed, with the aim to stimulate more extensive and in-depth investigations in improving electrochemical properties of LIBs via the advanced ALD nanotechnology. Even if tremendous achievements have been made with the ALD for rationally fabricating and/or optimizing electrode materials and inorganic SSE, a series of significant explorations need to be further attempted, and the underlying sur-/interfacial interaction mechanism should be clearly put forward. 

One challenging direction is to further develop some new ALD coating materials, which largely depends on the successful exploration of several novel precursors. The promising materials as surface-coating phases on electrodes should simultaneously afford fresh sur-/interfacial chemistry, unimpeded Li^+^ transportation and readily electrochemical reactions. As reviewed, Li-containing compounds, such as LiTi_2_(PO_4_)_3_ and Li_1.3_Al_0.3_Ti_1.7(_PO_4_)_3_ with high ionic conductivity (~10^−3^–10^−4^ S·cm^−1^), can be finely deposited with the existing precursors via the ALD for elegant surface modifications. Certainly, a considerable amount of other materials appropriate to ALD coating, such as metal oxides, fluorides, phosphates, and Li-containing compounds, should be persistently explored and optimized. It is especially worth mentioning that the surface modification of Ni-rich material (especially Ni content ≥ 0.6) is imperative. Unfortunately, modifying Ni-rich cathodes via the ALD strategy has still not been paid enough attention to fundamentally address the haunting sur-/interfacial issues.

Another promising direction would be to purposefully design and fabricate novel electrode material by the ALD for enhanced electrochemical activities, which can not only accommodate larger capacities, but also contribute to in-depth comprehension in the basics. It is well recognized that nanoscaled dimensions play a significant role in optimally approaching theoretical expectations of electrodes themselves, especially for those with poor ion/electron conductivity. As an advanced nanotechnology, the ALD is particularly suitable for precise growth of various thin films with controllable atomic thickness on bulk substrates, such as Si wafers. Nevertheless, it is necessary to further leverage the emerging electrode materials with varying scales and/or dimensions by exploiting novel ALD process. For instance, it is of vital significance to fabricate a series of olivine LiFe*_x_*Mn*_y_*PO_4_ cathode materials via the ALD in 2D and 3D carbonaceous substrates, thus considerably improving its rate capability.

Besides, it is also greatly meaningful to deeply unravel the intrinsic interaction mechanism between the ALD coating phases and bulk materials. Thus, it would be clarified how these ALD coating materials can substantially mitigate the degeneration of electrode materials by comprehensively understanding structural evolution with and without the ALD coating. Consequently, the exploration of the ALD combining with cathodes and anodes can be rationally predicted, and further validly implemented. Towards this goal, sophisticated characterizations, particularly in situ techniques, should be developed, and provide more mechanistic insights. 

Last but not least, with unique capability of the ALD to construct nanostructured films down to atomic scale, much more effort can be expected to build lithium-containing SSEs with high stability and exceptional ionic conductivity for all solid-state LIBs. More encouragingly, it is extremely interesting to construct an ideal heterostructure interface with low interfacial resistance via the ALD for inorganic SSEs application. 

In summary, the advanced ALD nanotechnology has been extensively demonstrated to tremendously propel the speedy advancement of high-performance LIBs. One should particularly note that as for the newly emerging fields, the employment of the appealing ALD in next-generation LIBs, and even in rechargeable Na-ion batteries, metal-air batteries, fuel cells, and so on, deserves more intensive yet extensive promotion in the future.

## Figures and Tables

**Table 1:** Summary of anodes, cathodes, and solid-state electrolytes (SSEs) fabricated via the atomic layer deposition (ALD) for lithium-ion batteries (LIBs).

Samples	Materials	Substrates	Precursors	Temperatures	Reference
Anodes	RuO_2_@LiPON	Si, MWCNT	C_14_H_18_Ru-O_2_ + LiO^t^Bu/H_2_O-TMP/N_2_	75 + 165 °C	[67]
	SnO_2_	Ni foam	TDMASn-H_2_O	150 °C	[68]
	SnO_2_	Graphene	Sn[(CH_3_)_2_N]_4_-H_2_O	150 °C	[69]
	SnO_2_	MXene + Cu	TDMASn-H_2_O	150, 200 °C	[70]
	SnO_2_	PAN	DBTDA/O_2_	100 °C	[71]
	SnO_2_@CoO	Ni foam	TDMASn-H_2_O	120 °C	[72]
	SnO_2_@TiO_2_ NTs	PAM	TDMASn-H_2_O	175 °C	[73]
	SnO_2_@TiO_2_	Carbon	SnCl_4_-H_2_O	400 °C	[74]
	TiO_2_	CNT@CFP	TiCl_4_-H_2_O	120 °C	[75]
	TiO_2_	Graphene	Titanium tetraisopropoxide-H_2_O	100 °C	[76]
	TiO_2_	Carbon film	Tetrakis dimethylamido titanium-H_2_O	200 °C	[77]
	TiO_2_	Carbon black	TiCl_4_-H_2_O	200 °C	[78]
	TiO_2_	CNTs	TiCl_4_-H_2_O	120 °C	[79]
	TiO_2_	Au	TiCl_4_-H_2_O	100–110 °C	[80]
	ZnO	Carbon black	Diethyl zinc-H_2_O	150 °C	[81]
	Li_4_Ti_5_O_12_	Graphene	TiCl_4_-H_2_O	120 °C	[82]
	WS_2_	Si, glass, SS	W(CO)_6_-H_2_S	175–205 °C	[83]
	MoN*_x_*	Si, glass, SS	Mo(CO)_6_-NH_3_	170 °C	[84]
	WN*_x_*	SS, CNTs	W(CO)_6_-NH_3_	180–195 °C	[85]
	LiTP	Si	Li(thd)/TPA	300 °C	[86]
Cathodes	V_2_O_5_	SS	VTOP/O_3_	170–185 °C	[87]
	V_2_O_5_	MWCNT, Si, SS	VTOP-H_2_O	120 °C	[88]
	V_2_O_5_	Si, glass, SS	VO(thd)_2_/O_3_	215 °C	[89]
	V_2_O_5_@TiO_2_	CNT	VOTP-H_2_O	150 °C	[90]
			TiCl_4_-H_2_O	120 °C	
	FePO_4_	NCNTs	(Fe(thd)_3_-O_3_) + (TMP-H_2_O/O_3_)	200–350 °C	[91]
	LiFePO_4_	Si, CNTs	5 × (FeCp_2_-O_3_-TMP-H_2_O) + (LiO^t^Bu-H_2_O)	300 °C	[48]
	LiCoO_2_	Si/SiO_2_, Si/TiO_2_/Pt	(CoCp_2_-plasmaO_2_) + (LiO^t^Bu-plasmaO_2_)	325 °C	[92]
	Li*_x_*Mn_2_O_4_	Si, SS	(Mn(thd)_3_-O_3_) + (Li(thd)-O_3_)	225 °C	[93]
SSEs	LiPON	Si	LiO^t^Bu/H_2_O + TMP	205 °C	[94]
	LiPON	Si(100) + borosilicate	LiN(SiMe_3_)_2_/H_2_NP(O)(OC_2_H_5_)_2_	270–310 °C	[95]
	Li_7_La_3_Zr_2_O_12_	Si(100)/MgO(100)	(LaFAMD/TDMAZ/TMA) + (LiO^t^Bu-O_3_)	200 °C	[96]
	Li*_x_*Al*_y_*Si_z_O	Si	LiOH/Al_2_O_3_/SiO_2_-H_2_O	290 °C	[97]
	Li*_x_*Al*_y_*Si_z_O	Si(100)	LiOC(CH_3_)_3_/Al(CH_3_)_3_/Si(OCH_2_CH_3_)_4_-H_2_O	225 °C	[98]
	Li*_x_*Ta*_y_*O_z_	Si	LiO^t^Bu-Ta(OEt)_5_-H_2_O	225 °C	[99]
	Li*_x_*Al*_y_*S	Cu	LiO^t^Bu/H_2_S(Al(N(C_2_H_5_)_2_)_3_)/H_2_S	150 °C	[100]
	Li_2_O-SiO_2_	Si, CNTs	LiO^t^Bu and TEOS	190 °C	[101]

multi-walled carbon nanotubes (MWCNT), trimethylphosphate (TMP), Tetrakis(dimethylamino) tin(IV) (TDMASn), Ti_3_C_2_T*_x_* (MXene), Polyacrylonitrile (PAN), Dibutyltindiacetate (DBTDA), Porous alumina membrane (PAM), Carbon nanotubes (CNTs), Stainless steel (SS), Li(thd) (thd = 2,2,6,6-tetramethyl-3,5-heptanedionate), benzene-1,4-dicarboxylic acid (TPA), vanadyl oxytriisopropoxide (VOTP), trimethylphosphate (TMP), trimethylaluminum (TMA), nanotube arrays (NTs), Solid-state electrolytes (SSEs), lithium tert-butoxide (LiO^t^Bu), Tris(*N*,*N*′-diisopropylformamidinato)lanthanum (LaFAMD), Tetrakis(dimethylamido)zirconium (TDMAZ), tris(2,2,6,6-tetramethylheptan-3,5-dionato) manganese(III)(Mn(thd)_3_), (2,2,6,6-tetramethylheptan-3,5-dionato)-lithium (Li(thd)), tetraethylorthosilane (TEOS).

**Table 2:** Summary of the ALD surface coatings on the anodes.

Materials	Coating Materials	ALD Precursors	ALD Temperature	Optimal Thickness, ALD Cycles	Reference
Carbon black	Al_2_O_3_	TMA-H_2_O	180 °C	~2 nm, 20 cycles	[143]
Carbon nanofibers	Al_2_O_3_	TMA-H_2_O	150 °C	10 cycles	[144]
Carbon	TiO_2_	TiCl_4_-H_2_O	120 °C	36.5 nm, 500 cycles	[104]
CNTs	FePO_4_@Li_3_PO_4_	C_10_H_10_Fe + C_3_H_9_O_4_P-H_2_O	300 °C	12 nm, 400 cycles	[145]
		C_4_H_9_LiO + C_3_H_9_O_4_P-H_2_O	250 °C		
CNTs	SiO_2_	APTES + BDEAS-H_2_O	150 °C	~10 nm, 300 cycles	[126]
Si	CNSs@Al_2_O_3_	TMA-H_2_O	150 °C	~6 nm, 50 cycles	[124]
Si	rGo@Al_2_O_3_	TMA-H_2_O	200 °C	~2 nm, 20 cycles	[125]
Si-nanowires	Al_2_O_3_	TMA-H_2_O	200 °C	~10 nm, 100 cycles	[129]
Amorphous Si	Al_2_O_3_	TMA-H_2_O	200 °C	~3 nm	[123]
Si	Al_2_O_3_	TMA-H_2_O	120 °C	~5 nm, 28 cycles	[132]
Si	TiO_2_	TTIP-H_2_O	150 °C	~3 nm	[130]
Li metal	Al_2_O_3_	TMA-H_2_O	180 °C	~2–3 nm, 30 cycles	[135]
Li metal	Al_2_O_3_	TMA-plasma O_2_	100 °C	~10.5 nm, 100 cycles	[134]
Li metal	Li*_x_*Al*_y_*S	Li_2_S-Al_2_S_3_	150 °C	~50 nm	[100]
Li metal	DOL@LiPON	LiO^t^Bu/TMP/N_2_-H_2_O	150 °C	~15 nm, 300 cycles	[127]
Ge	TiO_2_	TTIP-H_2_O	180 °C	~5 nm	[102]
SnS_2_	TiO_2_	TDMAT-H_2_O	150 °C	~4 nm, 80 cycles	[136]
SnS_2_	Al_2_O_3_	TMA-H_2_O	120 °C	~4.2 nm, 40 cycles	[137]
SnO_2_	GO@Al_2_O_3_	TMA-H_2_O	200 °C	~3 nm, 30 cycles	[138]
MoO_3_	HfO_2_	Hf(NMe_2_)_4_-H_2_O	180 °C	~3 nm, 10 cycles	[139]
Fe_3_O_4_	rGO@Al_2_O_3_	TMA-H_2_O	80 °C	~1 nm, 10 cycles	[140]
Fe_2_O_3_	TiO_2_	FeC_10_H_10_-H_2_O_2_	130 °C	~10 nm	[141]
CuO	Al_2_O_3_	Al(CH_3_)_3_-H_2_O	120 °C	~10–15 nm	[142]
Si-Cu-Ti	Cu_3_Si@Al_2_O_3_	TMA-H_2_O	250 °C	~2 nm, 20 cycles	[146]

Trimethyl aluminum (TMA), (3-aminopropyl)triethoxysilane (APTES), bis-(diethylamino)silane (BDEAS), Hollow carbon nanospheres (CNSs), titanium isopropoxide (TTIP), 1,3-dioxolane (DOL); tetrakis(dimethyl-amido) titanium (TDMAT); graphene oxide (GO); reduced graphene oxide (rGO); Tetrakis (dimethylamino) hafnium (Hf(NMe_2_)_4_).

**Table 3:** Summary of ALD surface coatings on the cathodes.

Cathodes	Coating Materials	ALD Precursors	ALD Temperatures	Optimal Thickness, ALD Cycles	Reference
Nano-LiCoO_2_	Al_2_O_3_	TMA-H_2_O	180 °C	~0.2 nm, 2 cycles	[147]
LiCoO_2_	TiO_2_/ZrO_2_/Al_2_O_3_	TTIP/Zr(NMe_2_)_4_/TMA-H_2_O	85/100/150 °C	~0.2–0.3 nm, 2 cycles	[118]
LiCoO_2_	AlF_3_	TMA-HF	150 °C	2 cycles	[148]
LiCoO_2_	AlW*_x_*F*_y_*	TMA-WF_6_	200 °C	~1 nm	[112]
LiCoO_2_	Al_2_O_3_/AlW*_x_*F*_y_*	TMA-H_2_O/TMA-WF_6_	150, 200 °C	~1 nm	[111]
SS LiCoO_2_	Li_2_PO_2_N	LiO^t^Bu + DEPA-H_2_O	140 °C	~90 nm	[149]
LiMn_2_O_4_	ZrO_2_	ZTB-H_2_O	120 °C	~1.2 nm, 6 cycles	[150]
LiMn_2_O_4_	CeO_2_	Ce(iPrCp)_3_-H_2_O	250 °C	~3 nm, 50 cycles	[151]
LiMn_2_O_4_	Al_2_O_3_	TMA-H_2_O	175 °C	~1 nm, 10 cycles	[152]
LiMn_2_O_4_	TiO_2_	TDMAT-H_2_O	150 °C	~5 nm	[153]
NMC333	Al_2_O_3_	TMA-H_2_O	180 °C	4 cycles	[154]
NMC333	LiTaO_3_	LiO^t^Bu + Ta(OEt)_5_-H_2_O	225 °C	5 cycles	[114]
NMC442	Al_2_O_3_	TMA-H_2_O	120 °C	0.6 nm	[155]
NMC532	Al_2_O_3_	TMA-H_2_O	120 °C	5 cycles	[156]
NMC532	Al_2_O_3_@Ga_2_O_3_	TMAl + TMGa-H_2_O + O_3_	200 °C	2 cycles	[119]
NMC532	MgO	Mg[EtCp]_2_-H_2_O	200 °C	~0.7 nm, 5 cycles	[157]
NMC811	LiAlF_4_	LiF@AlF_3_	250 °C	~25 nm	[113]
LiNi_0.5_Mn_1.5_O_4_	LiAlO_2_	TMA + LiO^t^Bu-H_2_O	200 °C	~2 nm, 10 cycles	[52]
LiNi_0.5_Mn_1.5_O_4_	FePO_4_	FeC_10_H_10_ + TMPO-H_2_O + O_3_	300 °C	~2 nm, 20 cycles	[158]
LiNi_0.5_Mn_1.5_O_4_	TiO_2_/Al_2_O_3_	TTIP/TMA-H_2_O	250 °C	0.0389/0.0816 nm	[159]
LiNi_0.5_Mn_1.5_O_4_	AlPO_4_	TMPO + TMA-H_2_O	250 °C	~1 nm, 10 cycles	[160]
Li_1.2_Ni_0.13_Mn_0.54_Co_0.13_O_2_	TiO_2_/Al_2_O_3_	TTIP/TMA-H_2_O	150 °C	~2–3 nm/2 nm	[161]
Li_1.2_Ni_0.13_Mn_0.54_Co_0.13_O_2_	AlPO_4_	TMPO + TMA-H_2_O	250 °C	~4 nm	[162]
Li_1.2_Ni_0.2_Mn_0.6_O_2_	Al_2_O_3_	TMA-H_2_O	150 °C	4 cycles	[163]

Titanium isopropoxide (TTIP), Solid State (SS), diethyl phosphoramidate (DEPA), Zirconium tert-butoxide (ZTB), tetrakis(dimethylamido) titanium (TDMAT), (CH_3_)_3_COLi (LiO^t^Bu), trimethyl gallium (TMGa), Bis(ethylcyclopentadienyl) magnesium (Mg[EtCp]_2_), LiF (LiO^t^Bu and TiF_4_) and AlF_3_ (AlCl_3_ and TiF_4_), trimethyl phosphate (TMPO), trimethyl phosphate (TMPO), LiNi_0.4_Mn_0.4_Co_0.2_O_2_ (NMC442).
